# Immunoscore Predicts Survival in Early-Stage Lung Adenocarcinoma Patients

**DOI:** 10.3389/fonc.2020.00691

**Published:** 2020-05-08

**Authors:** Zihuan Zhao, Dan Zhao, Ji Xia, Yi Wang, Buhai Wang

**Affiliations:** ^1^Department of Oncology, Subei People's Hospital of Jiangsu Province, Yangzhou, China; ^2^Dalian Medical University, Dalian, China; ^3^Department of Respiratory Disease, Nanjing Chest Hospital, Nanjing, China; ^4^Department of Reproductive Center, Zhen Jiang Fourth People Hospital, Jiangsu, China; ^5^Nanjing Medical University, Nanjing, China

**Keywords:** immunoscore, lung adenocarcinoma, prognosis, immune gene set, ridge regression

## Abstract

**Background:** The lung cancer staging system is insufficient for a comprehensive evaluation of patient prognosis. We constructed a novel immunoscore model to predict patients with high risk and poor survival.

**Method:** Immunoscore was developed based on z-score transformed enrichment score of 11 immune-related gene sets of 109 immune risk genes. The immunoscore model was trained in lung adenocarcinoma cohort from The Cancer Genome Atlas (TCGA-LUAD) (*n* = 400), and validated in other two independent cohorts from Gene Expression Omnibus (GEO), GSE31210 (*n* = 219) and GSE68465 (*n* = 356). Meta-set (*n* = 975) was formed by combining all training and testing sets.

**Result:** High immunoscore conferred worse prognosis in all sets. It was an independent prognostic factors in multivariate Cox analysis in training, testing and meta-set [hazard ratio (HR) = 2.96 (2.24–3.9), *P* < 0.001 in training set; HR = 1.99 (1.21–3.26), *P* = 0.006 in testing set 1; HR = 1.48 (1.69–2.39), *P* = 0.005 in testing set 2; HR = 2.01 (1.69–2.39), *P* < 0.001 in meta-set]. Immunoscore-clinical prognostic signature (ICPS) was developed by integrating immunoscore and clinical characteristic, and had higher C-index than immunoscore or stage alone in all sets [0.72 (ICPS) vs. 0.7 (immunoscore) or 0.59 (stage) in training set; 0.75 vs. 0.72 or 0.7 in testing set 1; 0.65 vs. 0.61 or 0.62 in testing set 2; 0.7 vs. 0.66 or 0.64 in meta-set]. Genome analysis revealed that immunoscore was positively correlated with tumor mutation burden (*R* = 0.22, *P* < 0.001). Besides, high immunoscore was correlated with high proportion of carcinoma-associated fibroblasts (*R* = 0.32, *P* < 0.001) in tumor microenvironment but fewer CD8+ cells infiltration (*R* = −0.28, *P* < 0.001).

**Conclusion:** The immunoscore and ICPS are potential biomarkers for evaluating patient survival. Further investigations are required to validate and improve their prediction accuracy.

## Introduction

Lung cancer ranks the top of cancer-related death worldwide (1). Histologically, 15 percent of patients are categorized as small cell lung cancer (SCLC) while the other 85% as non-small cell lung cancer (NSCLC) (2). Among NSCLC, lung adenocarcinoma (LUAD) is the most common subtype (3). Surgical resection remains to be the standard clinical practice for patients with early-stage LUAD (4), and the 5-year survival rate is about 60% (5). Platin-based adjuvant chemotherapy has demonstrated the improvement of 5-year survival for stage II–IIIA patients for about 5%, at the price of chemotherapy-induced toxicity (6, 7). Adjuvant immunotherapy with immune checkpoint inhibitors has come into several clinical trials, but no definitive effectiveness made so far (8). Although using the American Joint Committee on Cancer (AJCC) TNM staging system improves prognostic prediction, it is still inconclusive due to other unknown factors. Thus, the development of new biomarkers is imperative for stratifying risk and optimizing treatment for lung cancer patients with early stage.

Tumor immune microenvironment (TIM) has long been recognized as a crucial factor in cancer progression and metastasis (9). Several studies have explored the TIM as a prognostic biomarker in lung cancer (10). For example, Brambilla et al. found higher CD4+/CD8+ ratio conferred better survival in patients with NSCLC (11). Also, for cancer cell itself, programmed cell death protein ligand 1 (PD-L1) expression and tumor mutation burden (TMB) have been used to predict outcome in NSCLC patients. Several investigations have indicated that patients with high TMB or high PD-L1 expression were associated with poor survival in resected NSCLC patients and might benefit from adjuvant chemotherapy (12, 13). However, substantial patients with low PD-L1 expression and low TMB still have poor outcomes. Therefore, exploring additional prognostic markers based on TIM could benefit larger population (14).

In our research, we developed novel prognostic early-stage lung cancer immunoscore model by integrating enrichment score of 11 immune gene sets using ssGSEA algorithm. ssGSEA algorithm was based on gene ranks in and out of the selected gene set (15). To date several signatures used for phenotype classification or survival prediction have been developed by leveraging this algorithm (16–18). After immunoscore model construction in the training set, we evaluated its prognostic abilities in training, testing and meta-set. Moreover, we built Immunoscore-clinical prognostic signature (ICPS) by incorporating both immunoscore and clinical factors.

## Materials and Methods

### Clinical Data Processing

We used three largest publically available datasets, TCGA-LUAD, GSE31210, and GSE68465, deposited in Genomic Data Commons (GDC) portal (https://portal.gdc.cancer.gov) or Gene Expression Omnibus (GEO) website (https://www.ncbi.nlm.nih.gov/geo) (19–23). Clinical and pathological information regarding to TCGA-LUAD cohort were retrieved from cBioportal website (https://www.cbioportal.org) with “cdgsr” package (24–26), whereas information related to GSE31210 and GSE68465 were obtained through “GEOquery” package (27). Samples without overall survival (OS) information or with OS time of 0 were excluded. We also ruled out samples with documented neoadjuvant therapies to reduce potential confounding bias. TNM stage were used and transformed to AJCC staging groups. Samples with specific T subcategories (like T2a or T2b) were converted to staging groups according to AJCC 7th edition. T1N0, T2N0, T1N1, T2N1 or T3N0 were converted to stage 1A, 1B, 2A, 2B, respectively, conforming to AJCC 6th edition. For GSE31210 without TNM stage information, we used the pathological stage in the clinical file directly.

### RNA-seq and Microarray Data Preprocessing

Raw “.CEL” files of microarray data were downloaded from GEO website and read by “affy” package with the latest brainarray CDF files (October 2019, version 24) (28, 29). Robust multi-array average (RMA) algorithm in “affy” package was then applied to normalize gene expression intensity (28, 30). RMA algorithm included background adjustment, quantile normalization, and measurement summation when multiple probes were used to quantify the same gene expression intensity. After normalization, “arrayQualityMetrics” package was utilized to detect and exclude possible outliers (31). For RNA-seq data, level 3 FPKM data were downloaded using TCGAbiolink *R* package (32). FPKM values were then transformed into TPM values, which allowed a more direct comparison between samples as the sum of all TPMs in each sample were the same. As a result, the inflated statistical significance was reduced (33). TPM values were subsequently log 2 transformed to fit a more normal distribution. Entrez IDs were used across all platforms. Only samples with clinical information were retained. Finally, TCGA-LUAD cohort was used as the training set for immunoscore model construction, which contained 400 patients with RNA-seq data and survival information. Two microarray datasets, GSE31210 (*n* = 219) from Affymetrix Human Genome U133 Plus 2.0 Array platform as testing set 1, and GSE68485 (*n* = 356) from Affymetrix Human Genome U133A Array platform as testing set 2, were used to assess the immunoscore performance in predicting survival of early-stage LUAD patients.

### Immunoscore Construction

We searched Immport database (https://immport.niaid.nih.gov) and downloaded 1811 immune-related genes from 17 categories (18). Of 1,811 immune-related genes, 1,361 of them were contained in the training set. Univariate Cox proportional regression analysis was used to investigate their associations with patient survival using “survival” package (34). Only the genes with *P*-value < 0.05 and hazard ratio (HR) > 1 were screened out as immune risk genes for further study. We then implemented single sample gene set enrichment analysis (ssGSEA) algorithm to quantify the enrichment score of immune risk genes in various immune-related gene set using “GSVA” package (35). Difference of enrichment statistic of genes in the gene set and outside were computed, and normalized to fit a relatively uniform scale as Barbie et al. described (15, 35). We then transformed the normalized enrichment score into Z-score to conform standard normal distribution using the following algorithm:

$$\begin{array}{l} ZNES_{ij}=\frac{NES_{ij}-M_{j}}{SD_{j}} \end{array}$$

The final Z-score transformed normalized enrichment score of sample i, immune gene set j was denoted by *ZNES*_*ij*._ The normalized enrichment score of sample i, immune gene set j was denoted by *NES*_*ij*_. The mean and standard deviation (SD) of enrichment score across all samples in immune gene set j were denoted by *M*_*j*_ and *SD*_*j*_, respectively. This transformation obtained a uniform underlying distribution (mean = 0, standard deviation = 1) of each gene set across various platform; Immunoscore model was established by integrating all Z-score transformed normalized enrichment score using regularized Cox regression with the ridge penalty.

$$\begin{array}{l} {\text{Immunoscore}}_{\text{i}}=\sum_{\text{j}=1}^{n}\beta_{\text{j}}\;*\;{\text{ZNES}}_{\text{ij}}\; \end{array}$$

Immunoscore of sample i was denoted by *Immunoscore*_*i*._ Ridge Cox regression coefficient of gene set j was denoted by β_*j*_ and standard normal distribution transformed normalized enrichment score of sample i, immune gene set j was denoted by *SNES*_*ij*._ Ridge regression was used to address the possible collinearity (i.e., the correlated immune gene sets) to prevent overfitting (36). It was conducted by “glmnet” package and the tuning parameter Lambda was chosen with minimum criteria (37). Thus, a new variable immunoscore was created to predict patient survival. It could also be served as the quantitative measurement of hazardous level of tumor immune microenvironment with its biological background.

### Validation of Immunoscore

After immunoscore development, we applied the same formula to two independent testing sets, GSE31210 and GSE68485. Meta-set was formed by combining all training and testing sets. Univariate and Multivariate regression were used to evaluate the predictive power of the immunoscore model in all training, testing and meta-set. Age, stage, gender and smoking history were included in multivariable Cox analysis. Fraction of genome alteration in TCGA-LUAD clinical profile was also involved as a covariate in the TCGA-LUAD cohort. Patients were divided into high-immunoscore and low-immunoscore subgroups based on median value in the training set. Patients with immunoscore higher than cut-off value were assigned to high-immunoscore subgroup, while with immunoscore lower or equal to cut-off value were assigned to low-immunoscore subgroup. Kaplan-Meier analysis was performed to these two groups. Time-dependent receiver operator characteristic (ROC) curve analysis was utilized to assess the predictive accuracy for early-stage LUAD patients using “timeROC” package (38). The prognostic value of immunoscore in various treatment groups was evaluated in GSE68465, which contained detailed information of whether patients received adjuvant chemotherapy or radiotherapy with sufficient sample size in each category (75 patients with documented adjuvant therapy, 271 patients without documented adjucvant therapy).

### Comparison With Other Gene Expression Signatures

The immunoscore was compared with other existing NSCLC prognostic signature to assess its clinical utility. To date, numerous gene expression signatures have been developed. We selected two immune-related signatures (39, 40), one glycolysis-based signature. In addition, another malignancy gene signature was included, which had the top-ranked prognostic capability when compared with random signature in lung adenocarcinoma patient (41, 42). Detailed information regarding these signatures was provided in Supplementary Table 3. Gene symbols in the signatures were transformed into Entrez IDs. Using coefficients provided in supplementary materials, continuous risk score of each signature was computed in TCGA-LUAD, GSE31210, and GSE68465 cohorts. For malignancy gene signature, risk score was generated in each set by first principal component of provided gene list. Hazard ratios of univariate and multivariate Cox regression were used to evaluate their survival associations. C-index derived from “coxph” function with default Efron method to handle ties was utilized to determine their prognostic classification capabilities.

### Immunoscore-Clinical Prognostic Signature Construction

To make full use of both immunoscore and clinical variables in prognostic prediction, we constructed immunoscore-clinical prognostic signature (ICPS). Stage was converted to numeric variable. Stage IA, IB, IIA, IIB were assigned as 1, 2, 3, 4, respectively. Stage II with no subcategories were assigned as 3.5. Similarly, median value of ICPS in the training set was used as the cut-off value. C-index of ICPS was compared with stage or immunoscore alone using “compareC” package (43).

### Genomic Analysis

Somatic mutation profile were downloaded from Genomic Data Common (GDC) website. Maftools was used to summarize the somatic mutation (44). Samples measured by Whole Genome Amplification (WGA) of Repli-G DNA (which could be identified by tumor barcode) were excluded to reduce possible bias. Tumor mutation burden (TMB) was calculated as previous study described:

$$\begin{array}{l} {\text{TMB}}_{\text{i}}=1.0\;*\text{ NT}{\text{M}}_{i}+1.5\;*\text{ T}{\text{M}}_{\text{i}} \end{array}$$

Tumor mutation burden of sample i was denoted by TMB_i._ Total number of nontruncating mutation and total number of truncating mutation were denoted by NTMi and TMi, respectively.

The silent mutation was not included in the formula as it does not result in any change downstream. The truncating mutation was assigned a higher weight as it is considered more detrimental (45). Mutated genes between high-immunoscore and low-immunoscore groups were compared by fisher exact test using “mafcompare” function (44). Gene ontology and pathway analyses were then performed using differentially mutated genes by “clusterProfiler” package (46).

### Tumor Purity and Various Cell Composition Characterization

We established our immunoscore model based on the bulk gene expression data of the tumor. It could also be used as the measurement of hazardous level of tumor environment (TME) with its biological background. TME contained not only cancer cells, but surrounding non-cancerous immune and normal cells. To further delineate the correlation between immunoscore and TME, we need to first figure out the TME components. TCGA-LUAD cohort was used for TME evaluation. Tumor purity, the percentage of cancer cell inside the tumor, could be estimated in different ways. Aran et al. developed consensus measurement of purity estimations (CPE), which used the median value of several genomic algorithms and immunohistochemistry (IHC) after normalization by combined mean and standard deviation (47). As a result, the bias introduced by a single method or algorithm was minimized. We also utilized Estimating the Proportion of Immune and Cancer cells (EPIC) algorithm, a method to predict various cell types in tumor tissue using RNA-seq tumor gene expression profile (48). Non-log transformed TPM data of TCGA-LUAD samples were used and the Ensemble gene IDs were converted into the official gene symbols as the algorithm required. The EPIC algorithm was based on reference gene expression profiles to infer proportions of surrounding non-malignant cells with experimental measurements confirming its predictive robustness. Samples with convergence code other than 0 were excluded as these might be outliers.

### Gene Set Enrichment Analysis and Association With Clinical or Molecular Variables

Gene set enrichment analysis was performed to assess the association of immunoscore to the functional immune pathways. Differential gene expression profile between high-immunoscore and low-immunoscore subgroups was derived by “eBayes” function using limma package (49). We run fgsea algorithm with top 12,000 genes using C5 gene set from MsigDB database (https://www.gsea-msigdb.org/gsea/msigdb/). Gene set related to immune system were extracted. The correlation of immunoscore with clinical factors and certain molecular markers were also evaluated.

### Statistical Analysis

Group comparison between a continuous variable were conducted by *t*-test or ANOVA. All correlation analyses were performed with spearman method, and considered highly correlated when absolute value of correlation coefficient was >0.25. False discovery rate was calculated as the adjusted *P*-value. All statistical procedures were conducted by *R* software version 3.6.1 (50). All *p*-values were two-sided and considered statistically significant when <0.05. Gene set was with *P* < 0.05 and FDR < 0.25 was considered significantly enriched.

## Result

### Immunoscore Model Construction

**Figure 1:**
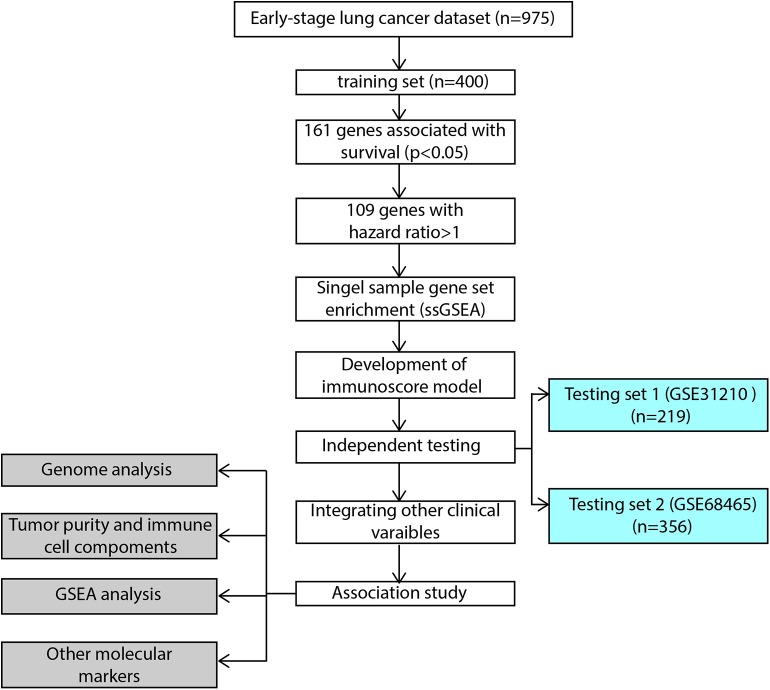
Flowchart of the study. GSEA, gene set enrichment analysis.

**Figure 2:**
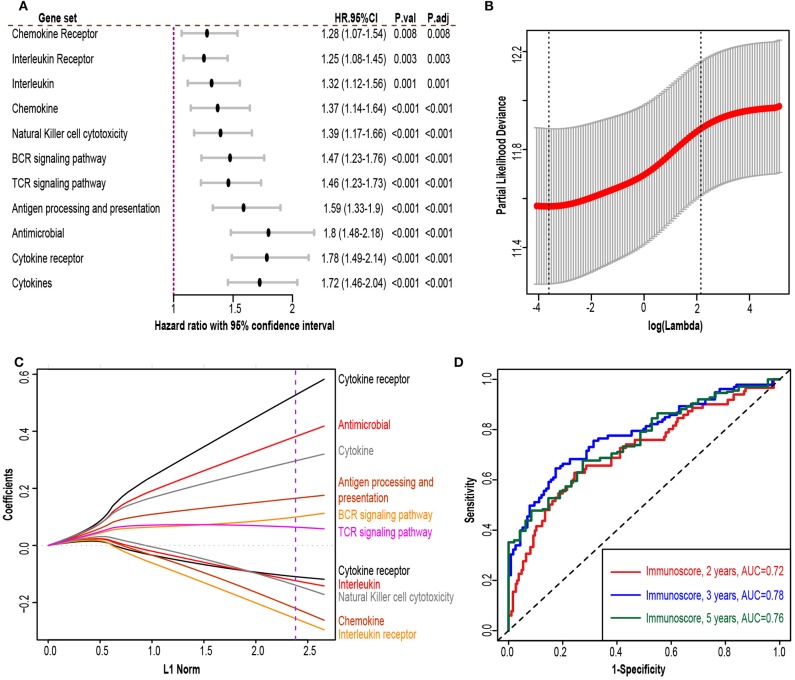
Immunoscore model construction. **(A)** Forest plot exhibiting different immune gene sets and patient overall survival in the training set. **(B)** 10-fold cross-validation for tuning parameter selection in the ridge regression model. The partial likelihood deviance is plotted against log (λ), where λ is the tuning parameter. Partial likelihood deviance values are shown, with error bars representing standard error (SE). The dotted vertical line on the left was drawn by minimum criteria whereas the line on the right represented the 1-SE criteria. We chose the minimum criteria. **(C)** Ridge regression coefficients of the 13 immune gene sets. The dotted line indicated the value chosen by the minimum criteria of the 10-fold validation. **(D)** Time-dependent receiver operator analysis (ROC) of the immunoscore in the training set. HR.95%CI, hazard ratio with 95% confidence interval. P.adj, adjusted *P*-value by false discovery rate.

The flowchart of our study procedures was illustrated in Figure 1. A total of 975 patients with early-stage lung adenocarcinoma were included in the study. Detailed clinical information was shown in Table 1. In the training set, 109 genes were correlated with worse prognosis (HR > 1, *P* < 0.05, Supplementary Table 1). Gene set “TGFb family members,” “TGFb_Family_Member” and “Interferons” contained only 1 gene and were excluded from further analysis. Z-score transformed enrichment scores of the remaining 11 gene set were then calculated as the method described. All of them were correlated with poor survival in the training set (Figure 2A; Supplementary Table 2). Ridge Cox regression was then performed and immunoscore was derived by the sum of all Z-score transformed enrichment scores weighed by ridge regression coefficients (Figures 2B,C; Supplementary Table 2). The predictive accuracy of immunoscore to 2, 3, and 5-year survival were estimated by time-dependent receiver ROC analysis (Figure 2D).

**Table 1:** Detailed patient clinical characteristics.

**Characteristics**	**Training set**	**Testing set 1**	**Testing set 2**
Source	TCGA	GSE31210	GSE68465
Sample size	400	219	356
Platform	RNA-seq	Affymetrix Human Genome U133 Plus 2.0 Array	Affymetrix Human Genome U133A Array
**AJCC stage**			
IA	130 (32.5)	112 (51.1)	112 (31.5)
IB	143 (35.8)	53 (24.2)	155 (43.5)
II	—	54 (24.7)	—
IIA	57 (14.2)	—	24 (6.7)
IIB	70 (17.5)	—	65 (18.3)
**Age group**			
≤ 65	190 (47.5)	170 (77.6)	186 (52.2)
> 65	201 (50.2)	49 (22.4)	170 (47.8)
**Smoking history**			
Non-smoker	57 (14.2)	112 (51.1)	40 (11.2)
Ever-smoker	333 (83.2)	107 (48.9)	243 (68.3)
Unknown	10 (2.5)	—	73 (20.5)
**Gender**			
Male	177 (45.6)	103 (46.4)	175 (49.2)
Female	211 (54.4)	119 (53.6)	181 (50.8)
**Survival status**			
Alive	273 (68.2)	186 (84.9)	189 (53.1)
Dead	127 (31.8)	33 (15.1)	167 (46.9)
**Genome alteration**			
≤ 0.2	170 (42.5)	—	—
> 0.2	229 (57.2)	—	—
Unknown	1 (0.2)	—	—

*Values in parentheses are percentages*.

### Validation of Immuoscore

**Figure 3:**
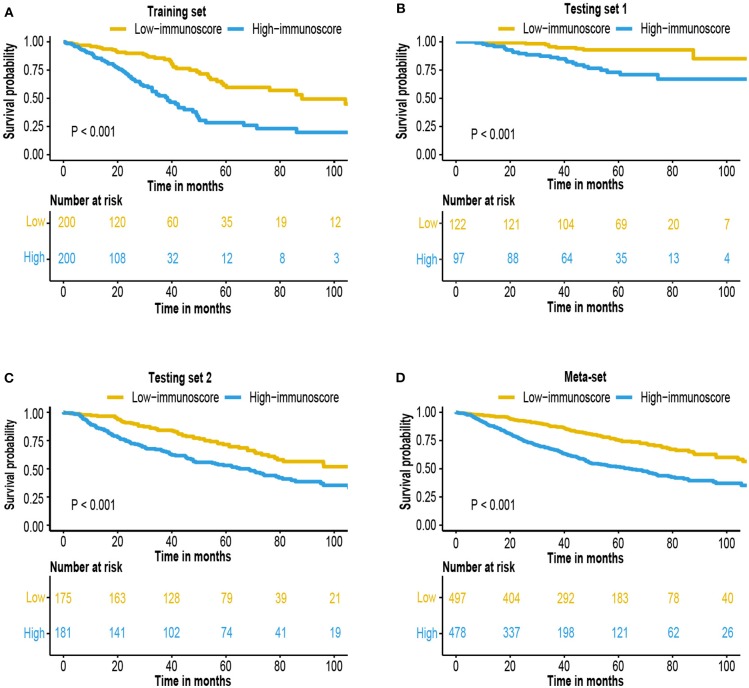
Survival analysis of the immunoscore. Kaplan–Meier curves for patient overall survival by immunoscore group in the **(A)** training set, **(B)** testing set 1, **(C)** testing set 2, and **(D)** meta-set.

The immunoscore of the testing sets were calculated using the same formula. We also built a meta-set by combing all training and testing sets. Patients were stratified into high and low-immunoscore subgroups using median value of immunoscore in the training set as the cut-off value (−0.0126). Kaplan–Meier survival analysis and log-rank test was performed to compare the difference between these two subgroups. The result exhibited that patients from high-immunoscore subgroup were more likely to suffer worse overall survival (*P* < 0.001 in the training set, testing sets, and meta-set; Figures 3A–D). Similarly, patients with higher score were also linked to shorter disease-free survival (DFS) interval (*P* < 0.001 in training set, testing set 1 and meta-set, *P* = 0.005 in testing set 2, Supplementary Figure 1). Time-dependent ROC analyses were also performed to testing sets and meta-set (Supplementary Figure 2).

**Figure 4:**
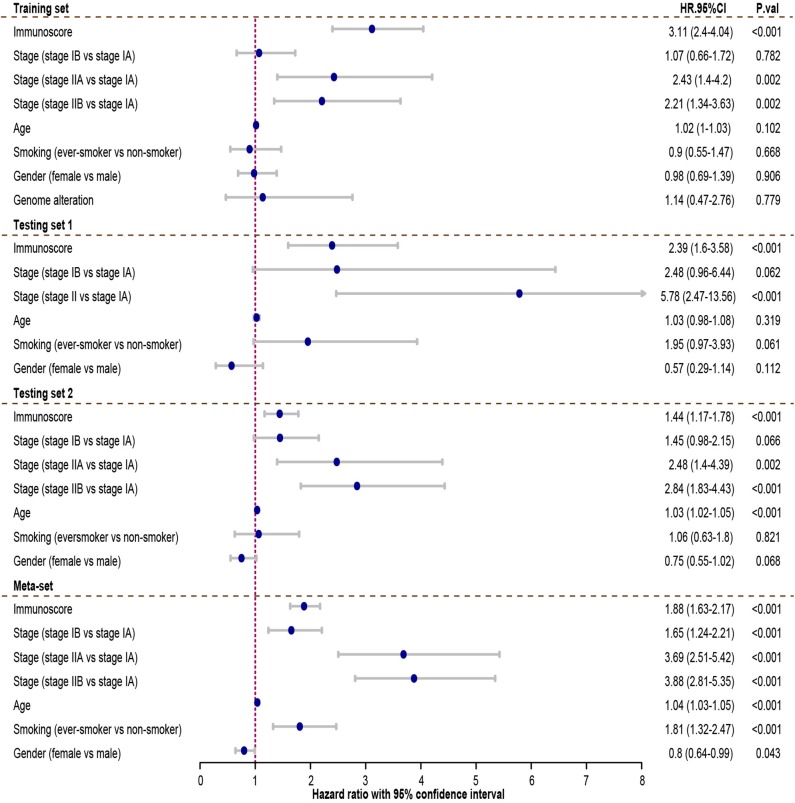
The univariate Cox analysis of the immunoscore and clinicopathological factors. The HR in training cohort was 3.11, with 95% confidence interval (CI) from 2.44 to 4.04 (*P* < 0.001). The HR in testing set 1 was 2.39, with 95% CI from 1.6 to 3.58 (*P* < 0.001). The HR in testing set 2 was 1.44, with 95% CI from 1.17 to 1.78 (*P* < 0.001). The HR in meta-set was 1.88, with 95% CI from 1.63 to 2.17 (*P* < 0.001). HR.95%CI, hazard ratio with 95% confidence interval.

**Figure 5:**
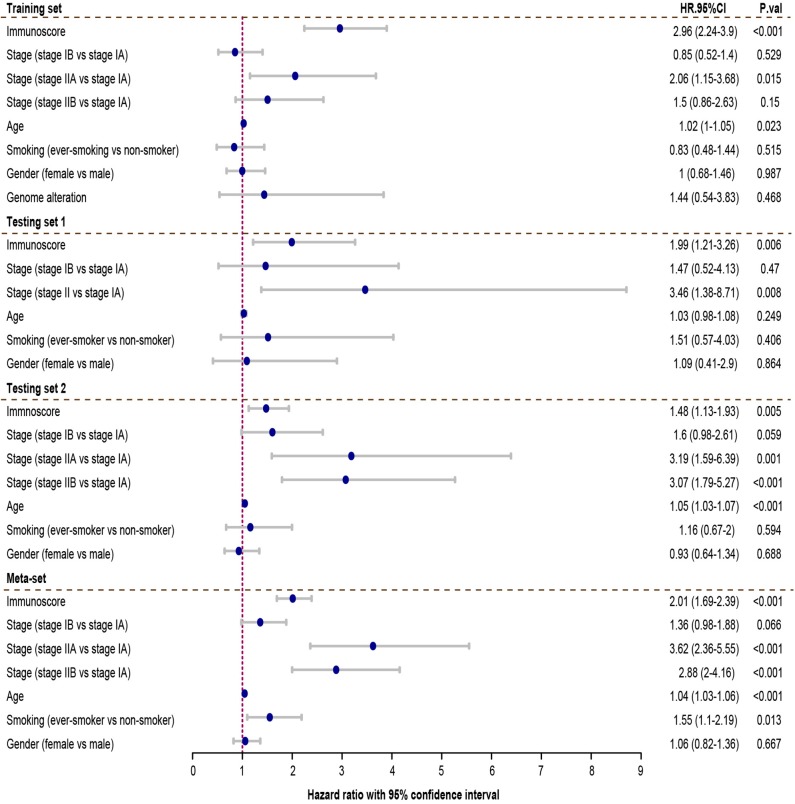
Multivariate Cox analysis evaluating independently predictive ability of immunoscore for patient survival. The immunoscore was able to independently predict patient survival in training set (hazard ratio (HR) = 2.96, 95% confidence interval (CI) from 2.24 to 3.9, *P* < 0.001), testing set 1 (HR = 1.99, 95% CI from 1.05 to 3.81, *P* = 0.006), testing set 2 (HR = 1.48, 95% CI from 1.13 to 1.93, *P*=0.005), and meta-set (HR = 2.01, 95% CI from 1.69 to 2.39, *P* < 0.001). HR.95%CI, hazard ratio with 95% confidence interval.

Cox regression was used to assess its survival association. Univariate Cox regression analysis revealed that immunoscore was a significant risk factor in all three training and testing sets (HR = 3.11, 95% confidence interval (CI) 2.4–4.04, *P* < 0.001 in training set; HR = 2.39, 95% CI 1.6–3.58, *P* < 0.001 in testing set 1; HR = 1.44, 95% CI 1.17–1.78, *P* < 0.001 in testing set 2; HR = 1.88, 95% CI 1.63–2.17 in meta-set; Figure 4). Multivariate Cox regression analysis indicated that immunoscore was an independent risk factor in training (HR = 2.96, 95% CI 2.24–3.9, *P* < 0.001), testing set 1 (HR = 1.99, 95% CI 1.21–3.26, *P* = 0.006), testing set 2 (HR= 1.48, 95% CI 1.13–1.93, *P* = 0.005), and meta-set (HR = 2.01, 95% CI 1.69–2.39, *P* < 0.001), as shown in Figure 5. Moreover, Immunoscore could identify patients with worse survival in all clinical subgroups in meta-set (Supplementary Figure 3).

### Comparison of Immunoscore With Other Genomic Signatures

**Figure 6:**
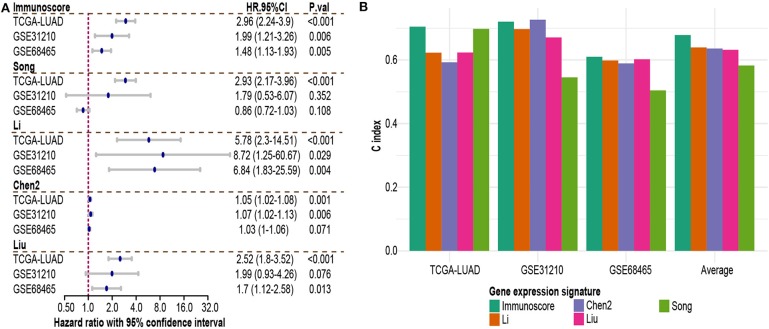
Comparison of immunoscore and other existing NSCLC signatures. **(A)** Hazard ratio of each gene expression signature in multivariable Cox analysis. **(B)** C-index of each signature in each independent dataset and mean C-index. HR.95%CI, hazard ratio with 95% confidence interval.

To assess the utility of immunoscore model, we compared prognostic association of immunoscore against other published genomic signatures (Supplementary Table 3). Besides Song et al. signature, most signatures had good performance in univariate and multivariate regression analyses (Supplementary Figure 4; Figure 6A). Immunoscore exhibited a generally higher C-index than other signatures in all three cohorts, except less than Chen2 et al. signature in GSE31210 (0.72 vs. 0.726, Figure 6B). Meanwhile, immunoscore achieved the highest mean C-index (0.68 vs. range from 0.58 to 0.64, Figure 6B).

### Immunoscore-Clinical Prognostic Signature Construction

Stage, age and immunoscore were all independent prognostic variables in multivariable Cox analysis in all 3 sets and meta-set. To explore whether combing these variables would improve prediction accuracy, coefficients of multivariate regression of these three factors in the training-set were used to introduce a new variable, immunoscore-clinical prognostic signature (ICPS).

$$\begin{array}{lll} ICPS & = & \;1.06076428\;*\;immunoscore\\ +0.19653598\;*\;stage+0.01961085*age \end{array}$$

**Figure 7:**
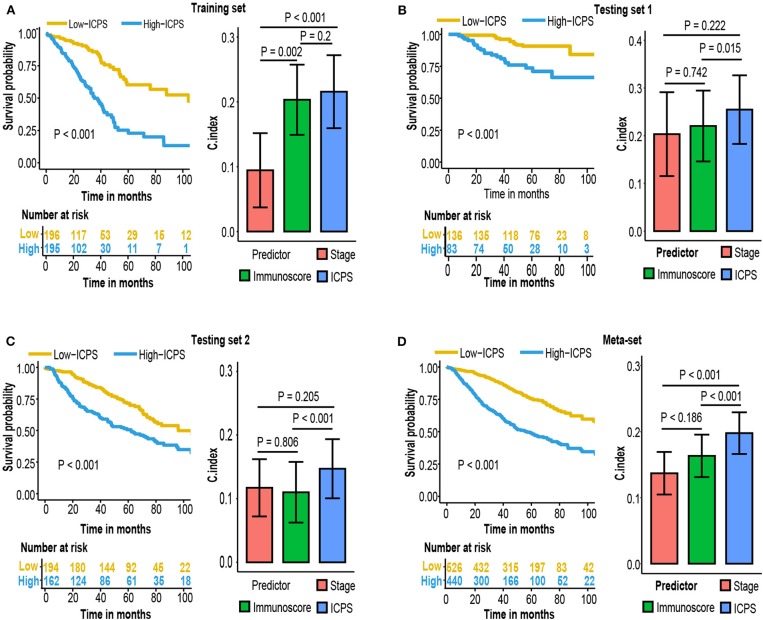
Kaplan–Meier survival analysis and compare C-index of ICPS with immunoscore and stage in **(A)** training set, **(B)** testing set 1, **(C)** testing set 2, **(D)** meta-set.

Patients were stratified into high-ICPS and low-ICPS subgroups using median value of ICPS in training set as the cut-off (1.74). High-ICPS subgroup was significantly correlated with worse survival in each set (*P* < 0.001, Figure 7). Figure 7 also exhibited C-index of ICPS was significantly higher than either immunoscore or stage, in training [0.72 (ICPS) vs. 0.7 (immunoscore) and 0.59 (stage), *P* < 0.001 when compared with stage], testing set 1 [0.75 (ICPS) vs. 0.72 (immunoscore) and 0.7 (stage), *P* = 0.015 when compared with stage], and testing set 2 [0.65 (ICPS) vs. 0.61 (immunoscore) and 0.62 (stage), *P* < 0.001 when compared with stage]. Moreover, C-index of ICPS was significantly higher than both of them in meta-set [0.7 (ICPS) vs. 0.66 (immunoscore) and 0.64 (stage), *P* < 0.001 when compared with immunoscore or stage, Figure 7].

### Immunoscore, ICPS, and Adjuvant Therapy

**Figure 8:**
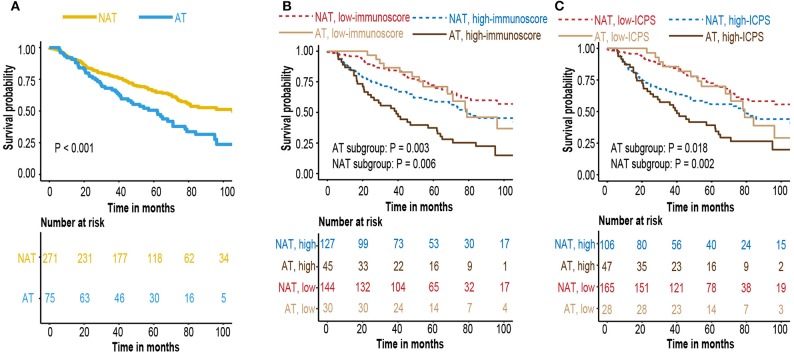
**(A)** Kaplan–Meier curves for patients who received adjuvant therapy or not **(B)** Kaplan–Meier curves for survival prediction by the immunoscore in patients who received adjunctive therapy or not. **(C)** Kaplan–Meier curves for survival prediction by the ICPS in patients who received adjunctive therapy or not.

A small subset of early-stage LUAD patient received postoperative adjuvant chemotherapy or radiotherapy. To investigate whether various treatment strategies had an effect to immunoscore and ICPS model, we used GSE68465 cohort with comprehensive documentation of adjuvant therapy. Patients who received adjuvant therapy had a worse overall survival (Figure 8A). It might be due to clinical practice, as adjuvant therapy was more likely to be applied to patients with higher stage and worse condition. Survival analysis indicated that immunoscore and ICPS could still stratify patients with different prognosis in each treatment group (Figures 8B,C).

### Genome Analysis

**Figure 9:**
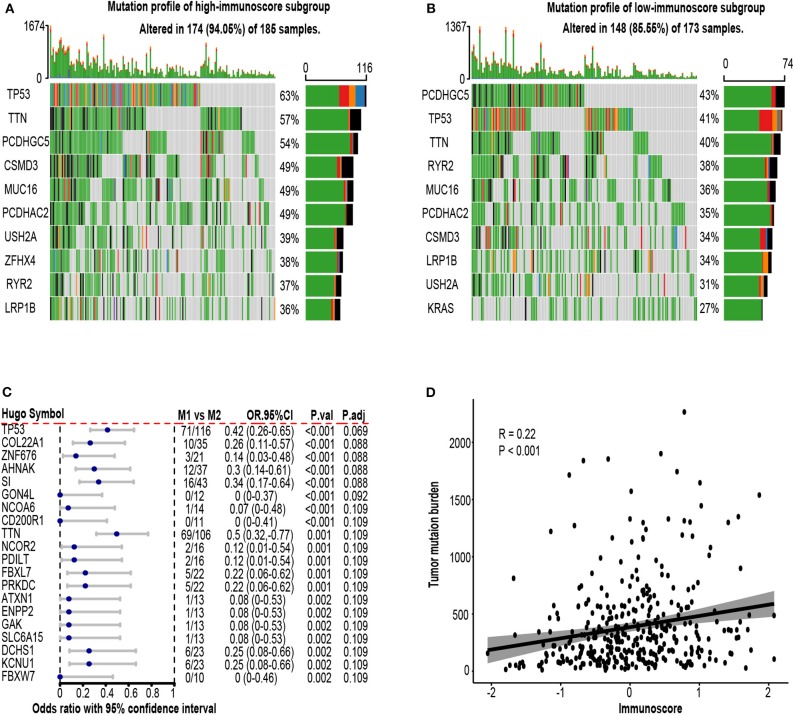
Mutation profile in the TCGA-LUAD cohort. **(A)** Mutation profile of high-immunoscore subgroup. **(B)** Mutation profile of low-immunoscore subgroup. **(C)** Differentially mutated genes between high and low immunoscore patients. **(D)** Correlation between tumor mutation burden (TMB) and immunoscore. OR.95%CI, odds ratio with 95% confidence interval. P.adj, adjusted *P*-value by false discovery rate.

To explore the possible underlying causes of difference in immunoscore between patients, we searched GDC website and downloaded all available somatic mutation data of lung adenocarcinoma patients. Three hundred fifty-eight available mutation profiles in TCGA-LUAD cohort (174 in high-immunoscore subgroup, 148 in low-immunoscore subgroup) were summarized by maftools. The mutation profiles of high-immunoscore and low-immunoscore subgroups were illustrated in Figures 9A,B, respectively. Differentially mutated genes between low-immunocore and high-immunoscore subgroups were identified by Fisher exact test using “mafcompare” function. Twenty of them were shown in Figure 9C. TP53 was the most commonly mutated gene in high-immunoscore subgroup and had the smallest adjusted *P*-value. TP53 was a tumor suppressor gene, encoding P53 transcriptional factor which responds to DNA damage repair. TP53 mutation has been recently reported to be associated with response to immunotherapy in certain subtype of NSCLC (51). We discovered that TP53 mutation was correlated with immunoscore. P53 mutation might induce genome instability, increasing neoantigen load, leading to a more dangerous tumor immune microenvironment, resulting in higher immunoscore. Another established immunotherapy biomarker, tumor mutation burden (TMB), was also positively correlated with immunoscore (*R* = 0.22, *P* < 0.001, Figure 9D). Gene ontology and KEGG pathway analyses of the differentially mutated genes were provided in Supplementary Figure 5.

### Immunoscore and Tumor Microenvironment

**Figure 10:**
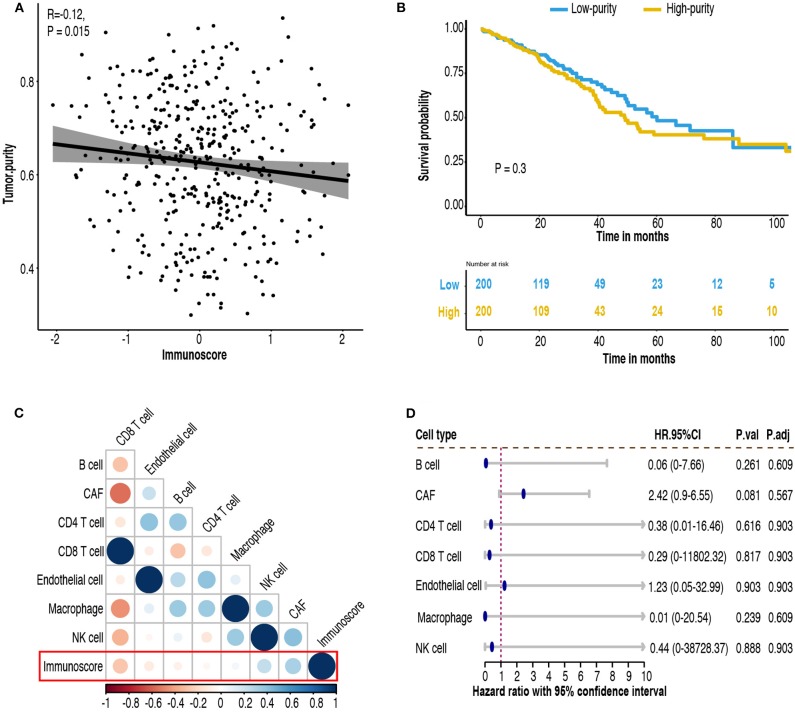
Tumor microenvironment (TME) change associated with immunoscore. **(A)** Correlation between immunoscore and tumor purity. **(B)** Kaplan-Meier curves of patient survival according to tumor purity in the TCGA-LUAD cohort. **(C)** Correlation matrix of immunoscore and cell proportions. **(D)** Univariate Cox analysis of various cell type. HR.95%CI, hazard ratio with 95% confidence interval. P.adj, adjusted *P*-value by false discovery rate.

The relationship between immunoscore and tumor microenvironment was investigated using TCGA-LUAD cohort. Tumor purity, the percentage of cancer cells inside the tumor, was estimated by consensus measurement of purity estimations (CPE). Patients with high immunoscore tend to have low tumor purity (*R* = −0.12, *P* = 0.015, Figure 10A). Patients were divided into high-purity and low-purity subgroup using median value of tumor purity (0.637). Kaplan-Meier survival curves indicated high tumor purity tend to have generally worse survival, but did not reach statistical significance (*P* = 0.3, Figure 10B). We next investigated the cellular composition of TME. EPIC algorithm, which was designed specifically for RNA-seq data, was used to infer the proportions of different infiltrating immune and stromal cells. Using absolute value of 0.25 as cut-off, cancer-associated fibroblast (CAF) (*R* = 0.32, *P* < 0.001) and CD8 T cell (*R* = −0.27, *P* < 0.001) were highly correlated to immunoscore (Figure 10C; Supplementary Table 4). In univariate Cox analysis, only CAF attained a borderline significant *P* value (HR = 2.42, 95% CI 0.9–6.55, *P* = 0.081, Figure 10D).

### Immunoscore, Clinicopathological Characteristics, and Biological Phenotypes

**Figure 11:**
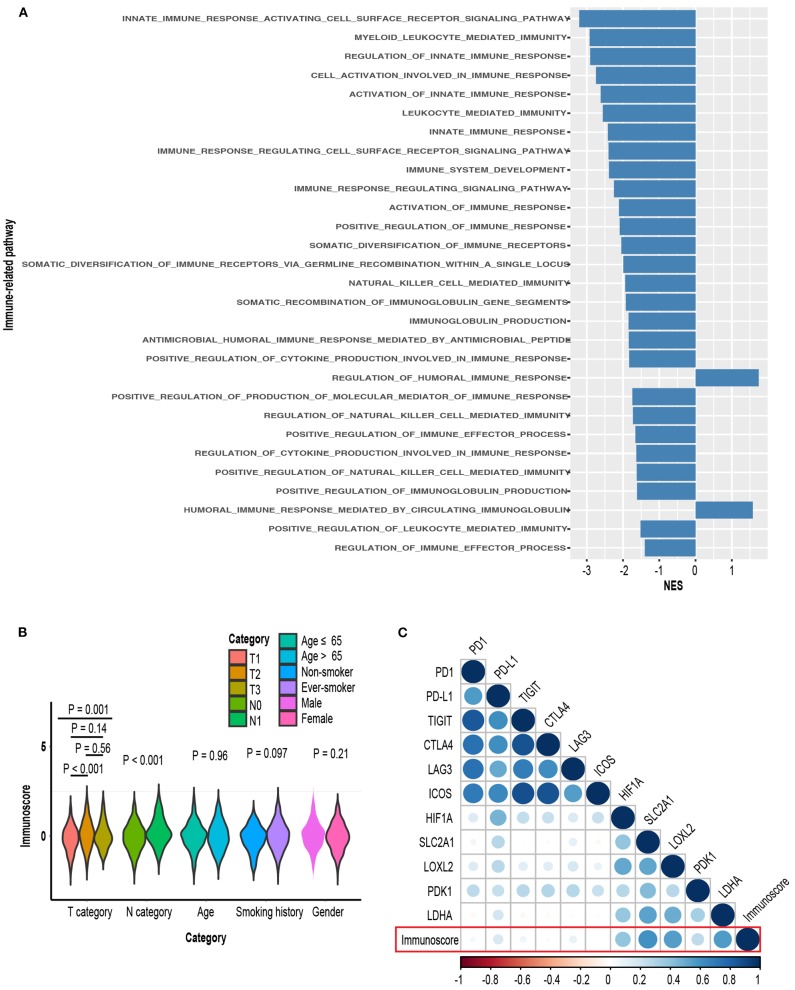
**(A)** Comparison of enrichment levels of immune-related pathways between high-immunoscore and low-immunoscore subgroups. **(B)** Distribution of immunoscore in various clinical subgroups. **(C)** Correlation matrix of immunoscore and certain gene expression.

Gene set enrichment analysis between high-immunoscore and low-immunoscore were conducted. Immune-related pathways were extracted and most of them were enriched to the low end (27 out of 29 Immune-related pathways). The relationship between immunoscore and other clinicopathological factors were assessed in TCGA-LUAD cohort. Higher T and *N* stage possessed greater immunoscore, whereas its distribution in age, gender and smoking status was not significantly different (Figure 11B). Of immune checkpoint molecules, immunoscore was only correlated to PD-L1 and LAG3 (*R* = 0.16, *P* = 0.001 for PD-L1; *R* = 0.1, *P* = 0.04 for LAG3, Figure 11B; Supplementary Table 5). Interestingly, Several hypoxia-inducible factor (HIF)-1 pathway markers, like HIF-1A (*R* = 0.41, *P* < 0.001), SLC2A1 (*R* = 0.6, *P* < 0.001), LOXL2 (*R* = 0.55, *P* < 0.001), PDK1 (*R* = 0.27, *P* < 0.001), and LDHA (*R* = 0.53, *P* < 0.001), were highly correlated with immunoscore (Figure 11C, Supplementary Table 5) (52).

## Discussion

Lung cancer treatment has been improved dramatically during the past decades, mainly owing to the constant discoveries of genomic alterations during lung cancer pathogenesis. However, the patient prognostic evaluation is still based on the AJCC staging system. Although it is a powerful prognostic prediction tool, it is inadequate to get a precise assessment of patient survival. In early-stage LUAD, the AJCC staging system is far from getting accurate prediction since about 30 percent of patients would develop recurrence, with 2-year survival at about 17% (53). To identify this subset of patients with high risk of recurrence and poor survival is critical since receiving adjuvant chemotherapy or newly developed adjuvant immunotherapy may of great benefit to them.

Up to now, Numerous gene expression signatures have been established for the prediction of lung cancer patient survival (41). Few of them, however, have been translated into real clinical practice. It might be caused by several defects in signature construction. First, some of them were trained from a small cohort with high variance and insufficient independent samples to test its robustness. Second, Gene expression data were measured by different experimental strategy with batch effect, which means that the signature constructed in one specific cohort cannot be generalized into other platforms. Third, most of the signatures were composed of several specific genes and ignore other possible causes, which severely decreased its stability and could potentially lead to overfitting.

In our research, we compared immunoscore with other gene expression signatures. Immunoscore achieved the highest mean C-index, indicating its superior prognostic classification capability. Of immune-related gene signatures, Li et al. (40) signature also had good performance. Li's signature used the binary variable, the pairwise comparison between immune-related gene groups, as features in model construction. Immunoscore and Li's signature had a lot in common, as both of them used some sort of gene ranks (ssGSEA in immunoscore; pairwise comparison in Li's signature) rather than gene expression intensity, making them not sensitive to preprocessing strategies and batch effect.

Our model also has its biomedical sense. It was constructed based on enrichment score of risk genes from multiple immune gene sets, and all selected immune gene sets were significantly correlated with worse patient survival. Thus, higher immunoscore indicated a more dangerous tumor microenvironment. The top three contributors to immunoscore were cytokine receptor, antimircrobial, and cytokine. Several cytokine-cytokine receptors signaling pathway have been identified to play a important role in cancer cell proliferation and survival. Most cytokine receptors were located at cell surface, and activated when contacting with specific cytokines. In GSEA analysis, innate immune response activating cell surface receptor signaling pathway ranked the top. Gene ontology analysis also indicated several gene sets related to cell membrane are enriched in differentially mutated genes. Overall, it implied that cell surface signaling pathways were tightly linked to immunoscore and disruption of these pathways might portend poor prognosis. In addition, drugs modifying cytokine-cytokine receptor signaling in combination with other immunotherapy might be a promising treatment strategy. Antimicrobial pathway has been linked to carcinogenesis, as infection by some microorganisms might lead to cell proliferation, and could be reversed by antimicrobials agents (54).

Tumor purity and cellular composition in tumor microenvironment were also investigated. Patients with high immunoscore tend to have low tumor purity. Furthermore, immunoscore was positively associated with CAFs and but inversely associated with CD8+ T cells. CD8+ T cell has direct cytolytic effect, whereas CAF, on the other hand, may suppress CD8+ cell function by upregulating PD-1, PD-L1, and FAS ligand on Treg cells (55). In addition, KEGG pathway analysis of differentially mutated genes also found ECM-interaction pathway abnormality. ECM stiffness might lead to activation of cancer cells and pro-tumor effect of CAF (56). Besides, cancer cell could induce CAF to remodel ECM, whereas CAF might sustain cancer growth by secreting aspartate (57). Further investigations are needed to figure out how fibroblast communicate with other cells or molecules inside TME and give insight to novel drug targets.

We next explored the phenotypical difference between samples of high and low immunoscore.

Most immune-related pathways were enriched in low-immunoscore subgroup, indicating high-immunoscore subgroup was a “immune cold” subtype. We also discovered multiple markers of HIF-hydroxylase oxygen-sensing pathway to be correlated with immunoscore. HIF could enhance tumor proliferation in TME by altering immune cell function and recruiting pro-tumor immune cells (58). For example, expression of HIF1A in tumor-associated macrophage (TAM) might suppress T cell function (59). More experiments and analyses are required to elucidate how HIF pathway affect tumor immune microenvironment as HIF1A is an incredibly promising target for cancer therapy (60).

Our study has several advantages. First, we trained our model in a large cohort with sufficient samples used to test its robustness. Second, we built our immunoscore model to predict patient outcome based on the enrichment levels of different gene sets rather than several single genes, making it a more comprehensive evaluation of tumor immune microenvironment and prevent overfitting. Third, when integrating clinical factors and immunoscore to construct a new ICPS model, it outperformed either immunoscore or stage alone. Fourth, immunoscore itself could also be seen as a proxy variable, the measurement of tumor immune microenvironment, and we found that genome instability, several specific immune cell proportions and functional pathway activation were correlated to immunoscore.

We admit some limitations. First, we used publically available datasets in retrospective manner. We did not have all clinical information needed for the study. For example, patients with inherent immune disorder or taking drugs with impact on immune system should be ruled out. Second, gene expression signatures were developed in different platform with diverse preprocessing strategies and normalization procedure. Although immunoscore outperformed other signatures, it might be due to technical bias or batch effect. Third, the immunoscore model contained several genes with still unknown effects in LUAD, and this “black-box” impact severely undermined the model interpretability. More experiments are needed to elucidate their biological associations. Finally, we cannot estimate its predictive value to immune checkpoint inhibitors due to lack of response data to immunotherapy. Further studies are needed to validate and improve immunoscore model.

## Data Availability Statement

Publicly available datasets were analyzed in this study. This data can be found here: https://portal.gdc.cancer.gov, https://www.ncbi.nlm.nih.gov/geo.

## Author Contributions

ZZ, YW, and BW were responsible for the study design. The analysis was performed by ZZ and DZ. ZZ and BW were involved in interpretation of the data. The manuscript was drafted by ZZ. JX and DZ have revised the manuscript. All authors read and approved the final manuscript.

## Conflict of Interest

The authors declare that the research was conducted in the absence of any commercial or financial relationships that could be construed as a potential conflict of interest.

## Supplementary Material

Supplementary Figure 1 Survival analysis of the immunoscore. Kaplan-Meier curves for patient disease-free survival by immunoscore group in the **(A)** training set, **(B)** testing set 1, **(C)** testing set 2, and **(D)** meta-set.

Supplementary Figure 2 Time-dependent receiver operator analysis (ROC) of the immunoscore in the **(A)** testing set 1, **(B)** testing set 2, and **(C)** meta-set.

Supplementary Figure 3 Subgroup analysis of immunoscore. Immunoscore was a significant risk factor in each clinical subgroup. HR.95%CI, hazard ratio with 95% confidence interval.

Supplementary Figure 4 Hazard ratio of each gene expression signature in univariable Cox analysis. HR.95%CI, hazard ratio with 95% confidence interval.

Supplementary Figure 5 Functional analysis of differentially mutated genes in genome analysis. Top 20 **(A)** biological process, **(B)** molecular function, **(C)** cellular component, and **(D)** KEGG pathway. KEGG, Kyoto Encyclopedia of Genes and Genomes.
