# Supplementary figures and images for: Immunoscore Predicts Survival in Early-Stage Lung Adenocarcinoma Patients
Source: Front Oncol. 2020 May 8;10:691. doi: 10.3389/fonc.2020.00691 (PMC7225293; doi:10.3389/fonc.2020.00691)

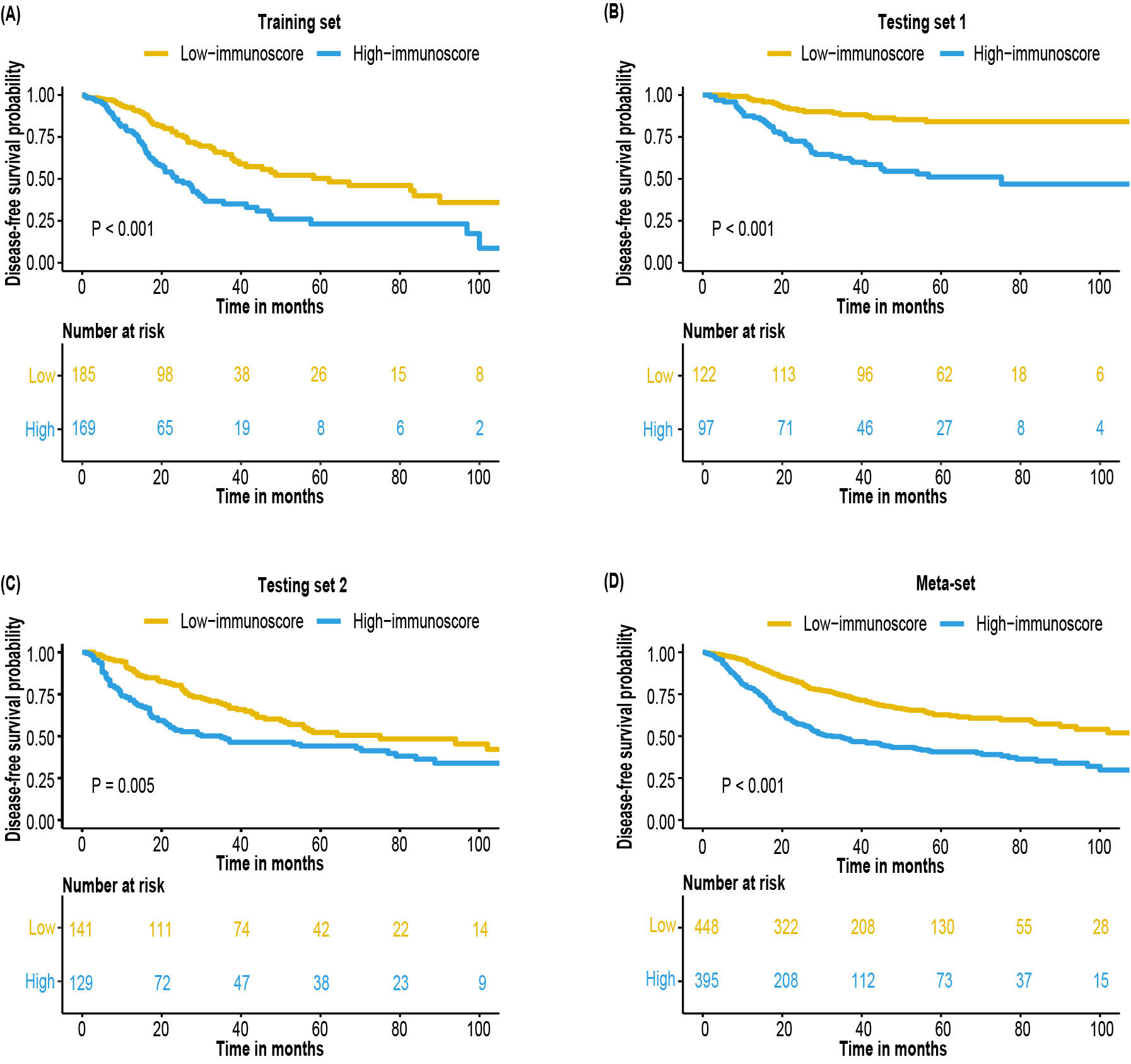

Supplement: Supplementary Figure 1 — Survival analysis of the immunoscore. Kaplan-Meier curves for patient disease-free survival by immunoscore group in the (A) training set, (B) testing set 1, (C) testing set 2, and (D) meta-set. [file Image_1.JPEG]

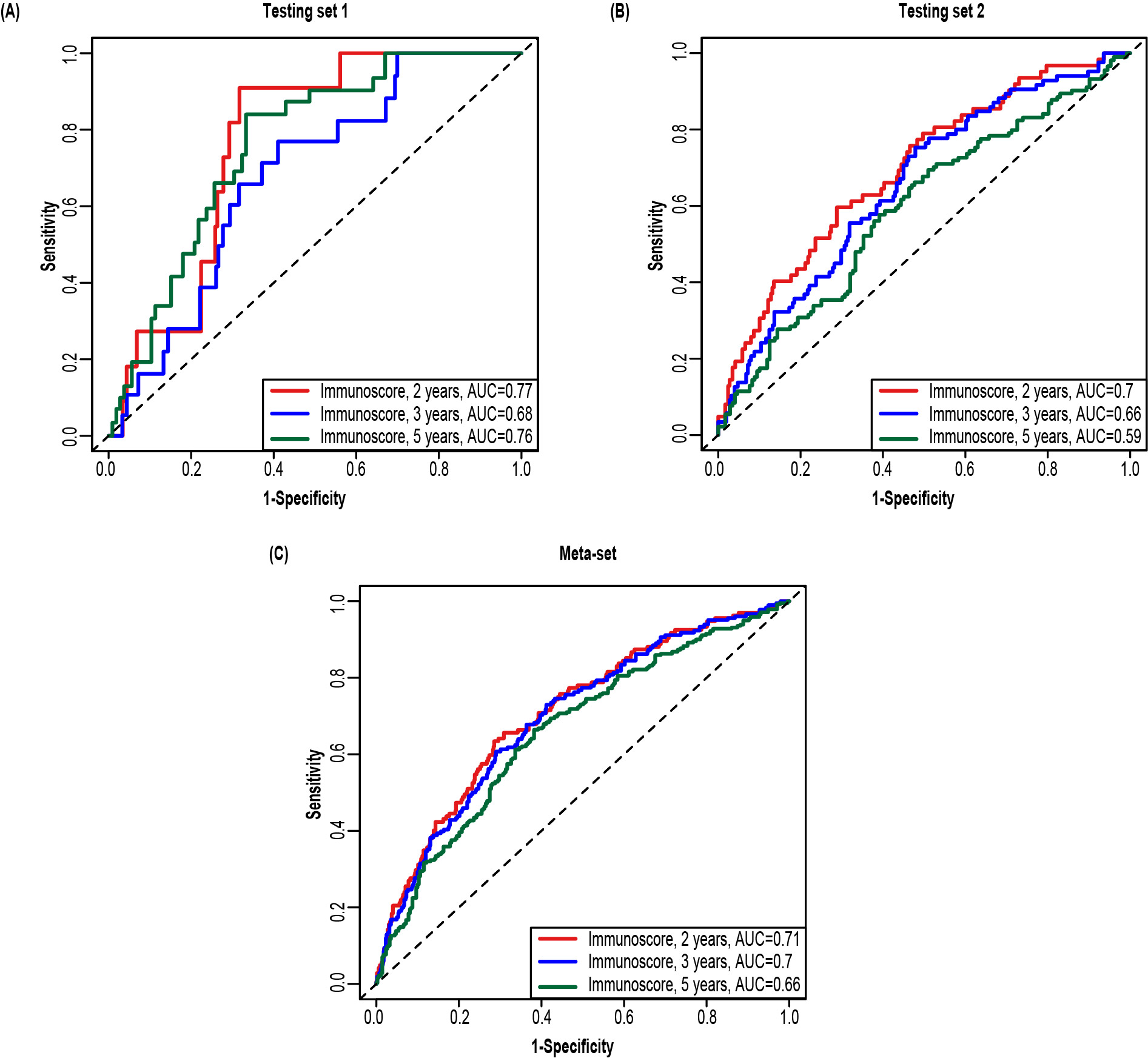

Supplement: Supplementary Figure 2 — Time-dependent receiver operator analysis (ROC) of the immunoscore in the (A) testing set 1, (B) testing set 2, and (C) meta-set. [file Image_2.JPEG]

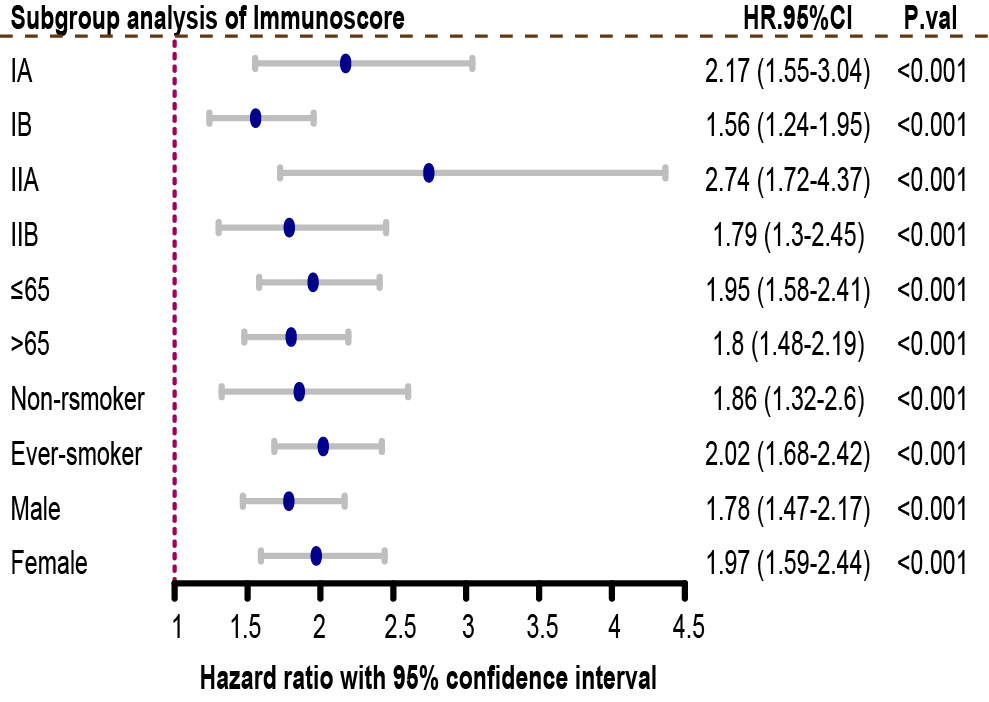

Supplement: Supplementary Figure 3 — Subgroup analysis of immunoscore. Immunoscore was a significant risk factor in each clinical subgroup. HR.95%CI, hazard ratio with 95% confidence interval. [file Image_3.JPEG]

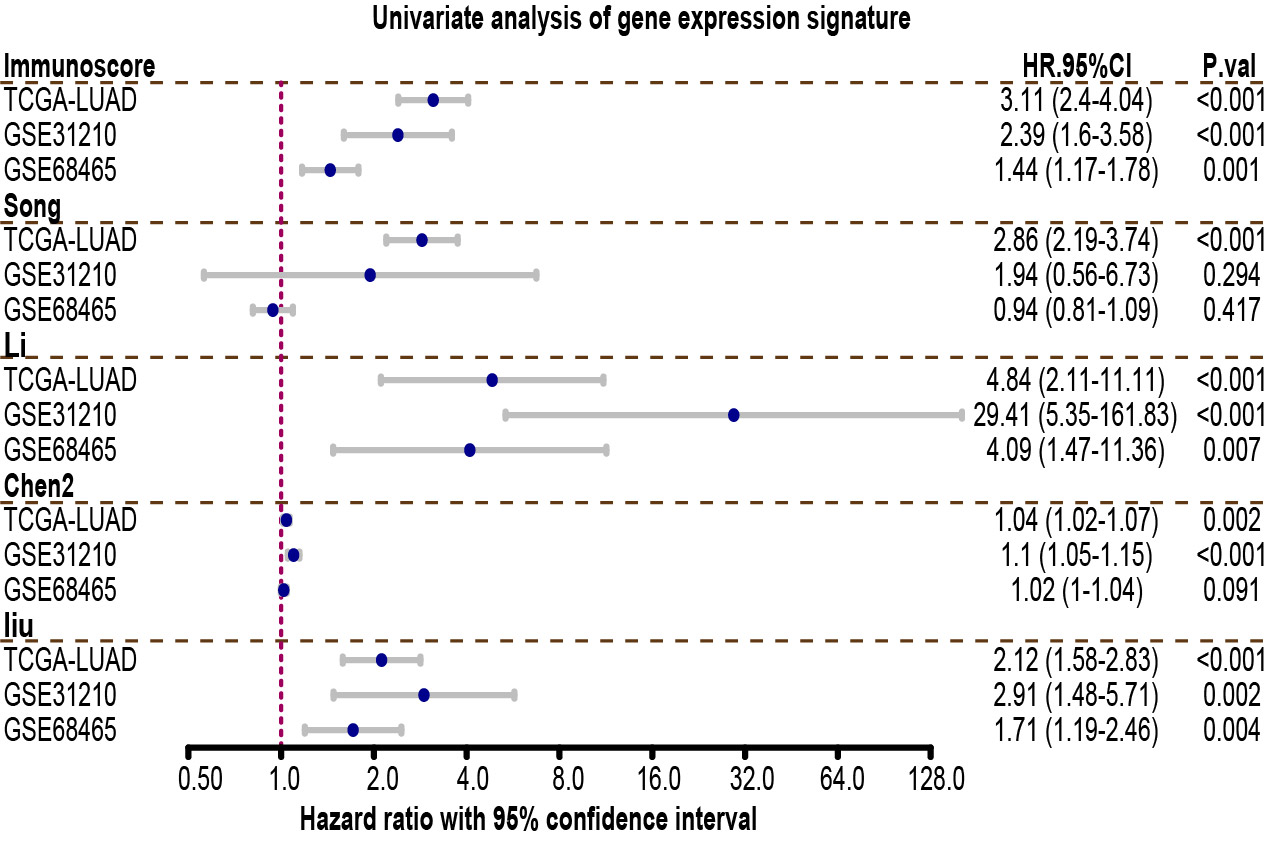

Supplement: Supplementary Figure 4 — Hazard ratio of each gene expression signature in univariable Cox analysis. HR.95%CI, hazard ratio with 95% confidence interval. [file Image_4.JPEG]

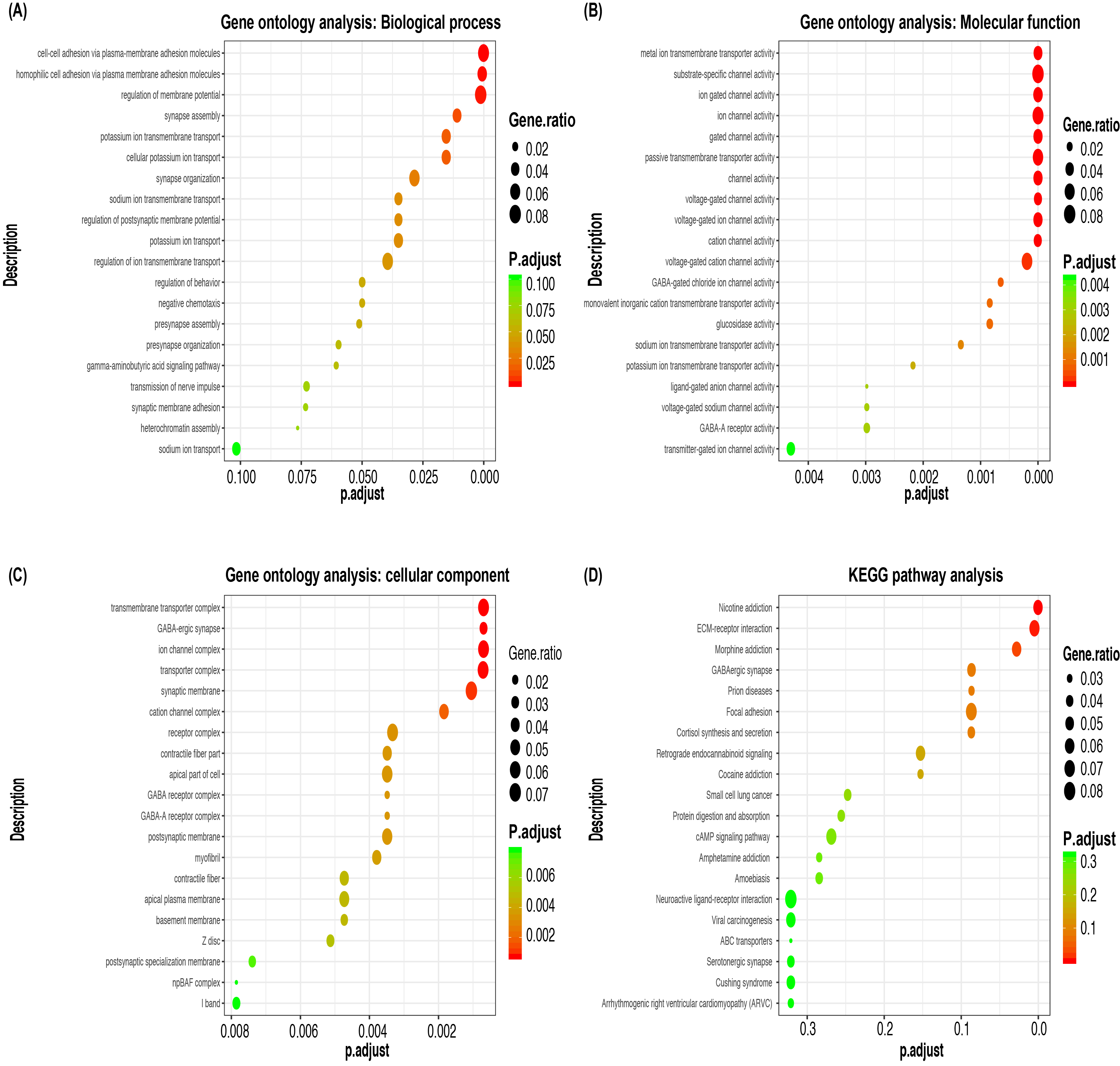

Supplement: Supplementary Figure 5 — Functional analysis of differentially mutated genes in genome analysis. Top 20 (A) biological process, (B) molecular function, (C) cellular component, and (D) KEGG pathway. KEGG, Kyoto Encyclopedia of Genes and Genomes. [file Image_5.JPEG]
